# Validation of an HPLC-DAD Method for Quercetin Quantification in Nanoparticles

**DOI:** 10.3390/ph16121736

**Published:** 2023-12-17

**Authors:** Daniel Carvalho, Ângelo Jesus, Cláudia Pinho, Rita Ferraz Oliveira, Fernando Moreira, Ana Isabel Oliveira

**Affiliations:** 1 Escola Superior de Saúde, Instituto Politécnico do Porto, Rua Dr. António Bernardino de Almeida, 4200-072 Porto, Portugal; 10170218@ess.ipp.pt (D.C.); acj@ess.ipp.pt (Â.J.); clp@ess.ipp.pt (C.P.); rfo@ess.ipp.pt (R.F.O.); aio@ess.ipp.pt (A.I.O.); 2 REQUIMTE-LAQV, Escola Superior de Saúde, Instituto Politécnico do Porto, Rua Dr. António Bernardino de Almeida, 4200-072 Porto, Portugal

**Keywords:** analysis, chromatography, HPLC, quercetin, validation

## Abstract

The evaluation of the efficacy of incorporation of quercetin in nanoparticles is crucial, both for the development and quality control of pharmaceutical formulations. The validation of analytical methods for the precise quantification of quercetin is useful for the evaluation of various potential quercetin delivery systems and quercetin pharmacokinetics. This work aimed to validate a high-performance liquid chromatography with diode array detection (HPLC-DAD) method for quercetin detection and quantification in nanoparticles. Different mobile phase conditions and detection wavelengths (254 and 368 nm) were tested, and the major validation parameters were assessed (precision, accuracy, linearity, sensitivity, stability, and selectivity). The best peak resolution was obtained when quercetin was analyzed at 368 nm with a mobile phase of 1.5% acetic acid and a water/acetonitrile/methanol ratio of 55:40:5. Under these conditions, quercetin also eluted rapidly (retention time of 3.6 min). The method proved to be linear (R^2^ > 0.995), specific, and repeatable (variation coefficient between 2.4% and 6.7%) and presented intermediate precision (variation coefficient between 7.2% and 9.4%). The accuracy of the analysis ranged between 88.6% and 110.7%, and detection and quantification limits were 0.046 and 0.14 µg/mL, respectively. Quercetin solutions were more stable when stored at 4 °C than at room temperature or −20 °C. This validated method satisfied more parameters of bias assessment than most recent methods for quercetin determination and presented itself as more sensitive and efficient than general spectrophotometric methods. The method was successfully used for the analysis of quercetin incorporation in nanoparticles and will be evaluated in the future for its adequacy for the determination of quercetin in more complex matrices.

## 1. Introduction

High-performance liquid chromatography (HPLC) is one of the most used chromatography techniques given its versatility, easy operation, and minimal solution preparation [1]. Additionally, unlike gas chromatography (GC), HPLC can be used for thermolabile and nonvolatile compounds that may be present in different matrices. Allowing both qualitative and quantitative approaches, HPLC enables the detection of several analytes from the same sample and the convenient adaptation of several chromatographic conditions [1,2].

UV/visible detectors are amongst the most used detectors in HPLC analysis, often in the form of a diode array detector (DAD). DAD enables simultaneous acquisition of the spectra of the peaks within a range of wavelengths. This feature enables the selection of the best-fitting wavelength, resulting in increased sensitivity [2,3].

All the analytical methods, whether newly developed or optimized, must go through a validation process for the compounds in the particular study to guarantee the reliability of results and ensure the scientific value of the developed method, assuring its reproducibility [4,5]. Several organizations have established guidelines indicating the validation process to be followed. The Food and Drug Administration (FDA), International Conference on Harmonization (ICH), and World Health Organization (WHO) are some of those examples. According to ICH, linearity, specificity/selectivity, limit of detection (LOD), limit of quantification (LOQ), precision, accuracy, robustness, and range of analysis are parameters that must be evaluated [6].

Quercetin, a flavonol, has received great attention in recent years due to its strong antioxidant [7], anti-inflammatory [8], cardiovascular [9], anticancer [10], and gastric protection [11] properties. It can be found in various fruits, vegetables, and plants [12,13] and has been shown to be effective in preventing and treating several cardiovascular diseases [9], neurodegenerative diseases [14], and cancer [10]. Quercetin is soluble in organic solvents, such as methanol (4.0 mg/mL, being moderately soluble at 37 °C) and dimethyl sulfoxide (150 mg/mL at 25 °C, presenting high solubility). On the other hand, it has low aqueous solubility (approximately 0.01 mg/mL at 25 °C) [15]. Despite its multiple potentialities for human health, its physicochemical properties, such as low bioavailability, easiest enzymatic degradation, and rapid metabolism, limit its application in the pharmaceutical industry [10]. Different attempts have been made to improve these properties using various methodologies [16,17].

Recently published reviews have depicted the sample preparation [18] and development and validation of analytical methods for the determination of quercetin from different matrices [19,20]. It was concluded that chromatography methods constitute the group of techniques most widely used for that purpose [19], while HPLC coupled with spectrophotometry detectors was the most popular method (surpassing mass spectrometry detectors). These findings suggest that most laboratories have conditions to analyze quercetin by HPLC-DAD and can benefit from the publication of optimized HPLC-DAD methods [20]. In one of the reviews, a risk-of-bias assessment with emphasis on method comparison was performed. The assessment was based on a previously published study that considered the evaluation of eight variables [21]. It was concluded that although all the included studies mentioned/presented totally or partially at least four of the eight parameters, only one of the studies fully satisfied more than four of the parameters (the study fully satisfied six of the parameters) [20]. These results further demonstrate the need for more complete and rigorous validation plans for quercetin determination.

To demonstrate improvements in biological effects, the efficient inclusion of quercetin in different distribution systems, and even the presence of quercetin in natural products, the validation of analytical methods that enable the detection and quantification of quercetin in the most varied matrices is crucial. Some of the important issues to be addressed include the full validation of methods that can be adopted in commonly existing equipment in most laboratories, such as HPLC-DAD; the characterization of parameters such as stability that has been scarcely depicted; and overcoming difficulties in distinguishing quercetin from structurally similar compounds. The main objective of the present study was to optimize and validate an HPLC-DAD method for quercetin detection and quantification for the analysis of quercetin in synthetic nanoparticles.

## 2. Results

**Figure 1:**
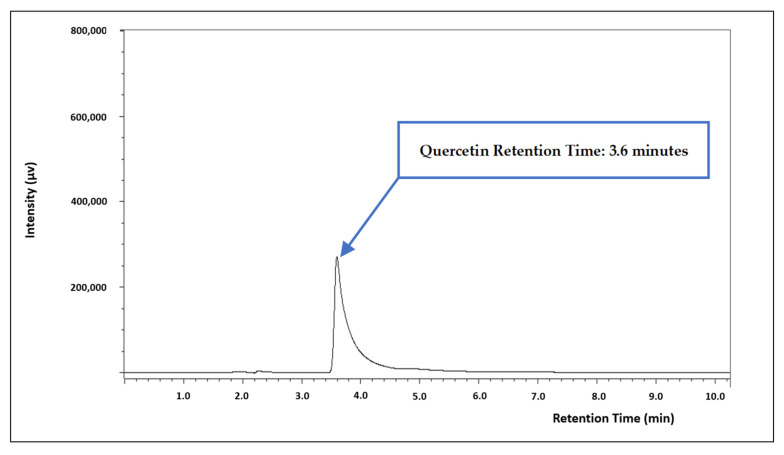
Chromatogram of quercetin (150 µg/mL) exhibiting the retention time at 3.6 min.

A higher chromatographic signal intensity was observed when the analysis took place at 368 nm than when the analysis took place at 254 nm. As for the mobile phase configuration, the ratio of 55:40:5 of water/acetonitrile/methanol, acidified with 1.5% acetic acid and with a variable flow rate between 1.0 and 1.3 mL/min proved to be the best option for quercetin quantification (Figure 1).

Regarding linearity, a calibration curve comprising nine standard concentrations was constructed. Additionally, two adjusted calibration curves comprising the five lowest concentrations of the linear range (adjusted calibration curve 1) and the five highest concentrations of the linear range (adjusted calibration curve 2) were also generated. The linearity of all three calibration curves was evaluated regarding the determination coefficient, the correlation coefficient, and their concordance after backcalculation. These results are depicted in Table 1 and Table 2. Figures S1 and S2 provided in the Supplementary File exhibit the obtained calibration curves, and Figures S3 and S4, also in the Supplementary File, present the difference plots.

The linearity of all three calibration curves and their respective equations was additionally determined with the intercept of the ordinate at origin and relative standard deviation (RSD) of the slope criteria, as shown in Table 3.

As for sensitivity determination, an LOD of 0.046 µg/mL and a LOQ of 0.14 µg/mL were obtained.

Intra- and interday precision results are presented in Table 4. Regarding repeatability, RSDs were equal to/lower than 6.74%, whereas intermediate precision presented a variation equal to/lower than 9.42%.

The accuracy of the method was determined relative to the adjusted equations. The concentrations of 0.35, 0.49, and 0.57 µg/mL, relative to the adjusted Equation (1), resulted in accuracies between 88.6% and 104.1%. The concentrations of 49, 125, and 196 µg/mL, relative to the adjusted Equation (2), resulted in accuracies between 96.8% and 110.7% (Table 5).

To estimate the specificity of the method, besides quercetin, mixed solutions containing rutin and kaempferol were also analyzed. Rutin presented a retention time of 2.5 min and kaempferol a retention time of 5.4 min. Both chromatographic peaks were visually distinct from the quercetin chromatographic peak, enabling their identification and quantification.

After promoting a slight variation in optimized chromatographic parameters, namely, pH of the mobile phase (+0.11) and flow rate (+0.2 mL/min), it was possible to observe a diminishing of the quercetin peak areas (Table 6).

The results of stability studies of quercetin after 5 and 7 days of storage under different conditions (−20 °C, 4 °C, and room temperature) are depicted in Table 7.

## 3. Discussion

### 3.1. Chromatographic Conditions

DAD detectors enable the evaluation of a wide range of wavelengths in the UV and visible region (190–900 nm). Accordingly, the most suitable wavelength can be chosen for the analyte under study [2]. The two most commonly studied wavelengths for quercetin detection and quantification (254 [22,23] and 368 [24] nm) were evaluated. The higher chromatographic signal intensity at 368 nm is related to the quercetin molecular structure, more precisely to its B and C rings and hydroxyl groups, which demonstrate an intense absorption in a wavelength range between 300 and 400 nm due to electron transitions [25,26].

Previously published methods developed by Aguilar-Sánchez et al. [27], Ang et al. [28], Antal et al. [29], Sree et al. [30], and Sousa et al. [31] used more distinct columns and detectors to those available in our laboratory to perform the analysis. Therefore, by choosing the method developed by Zu et al. [24] to test initial conditions, there was a higher probability of more efficient implementation. After verifying a pronounced tailing phenomenon with the adoption of the mobile phase composition described in the study by Zu et al. [24] (consisting of a mixture of water, acetonitrile, and methanol (45:15:40) in an isocratic elution and at a flow rate of 1.0 mL/min), different conditions were tested. Accordingly, the percentage of water was tested between 45% and 70% (increases of 5%), the percentage of acetonitrile was tested between 15% and 40% (increases of 5%), and the percentage of methanol was tested between 0% and 40% (increases of 5%). Acetic acid was included at concentrations of 0%, 0.5%, 1.0%, and 1.5%. The tested flow rate conditions varied between 1.0 and 1.5 mL/min, and both isocratic and gradient conditions were tested. The chromatographic condition that ensured the biggest reduction in peak tailing was considered as the primary criterion for the choice of mobile phase. The secondary criteria was the least consumption of organic solvents (for economic and ecological purposes). As previously mentioned, a ratio of 55:40:5 of water/acetonitrile/methanol, acidified with 1.5% acetic acid and with a variable flow rate between 1.0 and 1.3 mL/min proved to be the best option for quercetin quantification.

### 3.2. Method Validation

#### 3.2.1. Linearity

This method was tested for linearity using linear regression and correlation coefficient. Guidelines define that a minimum of five concentration levels should be analyzed to construct a calibration curve, along with three analyses. This was accomplished in this case because nine standard concentrations were used in triplicate [6].

A linear regression was observed between the peak area and analyte concentrations after visual inspection, ranging between 0.14 and 245 µg/mL with a correlation coefficient of 0.999, thus proving the method’s linearity. It is generally assumed that the method is more linear the closer the correlation coefficient is to 1 [4,32]. Although linearity was proven for the referred concentration range, a subdivision of the calibration curve was performed. When the curve standards were backcalculated and the respective deviations were analyzed, there was a reduction in the deviations of the adjusted equation in comparison to the unadjusted equation (Table 2). Thus, to achieve the most accurate quantification as possible and cover a broad linearity range of concentrations, it was necessary to divide the calibration curve into two, with each one containing five points, resulting in a calibration curve with a linearity range for lower concentrations (0.14 to 5 µg/mL) and another for higher concentrations (5 to 245 µg/mL). This demonstrated the suitability of the method for estimating a concentration of 5 µg/mL and encompassing a larger range than the studies of Abdelkawy et al. [22] (linearity range: 0.10–25 µg/mL), Olszewska [33] (0.1–77.92 µg/mL), Ang et al. [28] (1.25–200 µg/mL), Antal et al. [29] (6.0–192 µg/mL), Careri et al. [34] (0.45–57.60 µg/mL), Chen et al. [35] (1.11–22.10 µg/mL), Zu et al. [24] (19–280 µg/mL), Zhang et al. [36] (10,000–350,000 µg/mL), and He et al. [37] (14–70 µg/mL). This broad range of concentrations is particularly useful in cases in which there is no estimated concentration of quercetin.

However, some authors consider that the correlation coefficient is not the most appropriate parameter for linearity determination [38]. Therefore, linearity was also determined using the intercept of the ordinate at origin and RSD of the slope criteria [6,38].

After analysis of the results (Table 3), it can be concluded that both criteria were satisfied. According to the intercept of the ordinate at origin criterion, zero should be contained in the ordinate at the origin, and this was verified in all the curves [6,38]. According to the slope RSD criterion, the standard error of the slope should be less than 5%, which was also checked, and the errors were between 1.38% and 3.93% [38]. Thus, it can be stated that a strong linearity relationship was observed in all the calibration curves.

#### 3.2.2. Detection and Quantification Limits

The obtained LOD and LOQ values (0.046 and 0.14 µg/mL, respectively), along with the calibration curve slope, showed the high sensitivity of the analytical method in question for concentration values in the µg/mL range. In addition, the value found for LOQ was concurrent with the concentration of the lowest standard in linearity. Accordingly, it may be affirmed that it is possible to analyze quercetin accurately and precisely in samples with analyte concentrations within the determined linearity range. The evidenced sensitivity may be considered noninferior to the previously published methods on quercetin detection by Zu et al. [24] (LOD = 3.0 µg/mL), Choudhari et al. [39] (LOD = 0.66 µg/mL, LOQ = 2.0 µg/mL), and Abdelkawy et al. [22] (LOD = 0.05 µg/mL, LOQ = 0.1 µg/mL).

#### 3.2.3. Precision

Regarding precision, the guidelines recommend that at least three concentrations and a minimum of nine measurements covering the linearity range should be assessed and determined by the RSD [6,32]. In this case, precision was evaluated for five concentration standards with 15 measurements each (five samples/independent tests in triplicate), covering the entire range and including the orders of magnitude. Concentrations of 0.35 and 0.57 µg/mL were deemed relevant for the adjusted calibration curve 1, whereas concentrations of 125 and 185 µg/mL were relevant for the adjusted calibration curve 2. Because the 5 µg/mL concentration was included in both the linear ranges of calibration curves 1 and 2, its evaluation of precision was considered adequate for both ranges. The RSD of repeatability showed values ranging from 2.71% (125 µg/mL) to 6.74% (5 µg/mL), while the RSD of intermediate precision varied between 6.87% (5 µg/mL) and 9.42% (0.35 µg/mL), revealing a slight positive tendency (increased precision came with an increased standard concentration) [40]. According to the guidelines, RSD should be less than 15% for each standard concentration tested, which was verified in the optimized method, confirming the method’s repeatability and intermediate precision and showing that although small variations in analytical method conditions may occur, precise quantification is achievable [32,40].

#### 3.2.4. Accuracy

The closeness of the obtained value after chromatogram analysis with the real value determines the accuracy, and it should be established throughout the linearity range as it is one of the most important parameters to be evaluated [6,32]. This parameter could be studied by the application of different techniques, in which case, a reference standard is used. Guidelines define that the determination of accuracy should follow a minimum number of analyses and tested concentrations equivalent to that defined for precision, as previously mentioned [4,6]. For accuracy, six levels of concentrations were tested in triplicate. The three lower concentrations (0.35, 0.49 µg/mL, and 0.57 µg/mL) were considered to study the accuracy of the adjusted calibration curve 1, whereas the three higher concentrations (49, 125, and 196 µg/mL) were included to study the accuracy of the adjusted calibration curve 2. Accuracy should be equal to or lower than 15% variation for every concentration level tested, that is, it should be between 85% and 115% [32].

Conveniently applying the most adequate curve equation according to the expected concentration, the accuracy ranged between 88.6% (0.35 µg/mL) and 110.7% (196 µg/mL), with the results compiled in Table 5.

#### 3.2.5. Specificity/Selectivity

To ensure that it would be possible to distinguish quercetin from similar compounds, two other polyphenolic compounds [6] whose chromatographic separation from quercetin is considered to be challenging were selected (rutin and kaempferol) [41]. Both rutin and kaempferol exhibit the same backbone as quercetin; however, kaempferol does not present a hydroxyl group in the C3’ position [42], and in the case of rutin, a sugar group is present in the C3 position [43]. From the results, it is possible to infer that the optimized method demonstrates good selectivity as a clear separation of the three compounds was verified with good resolutions, which also suggests the method’s accuracy, precision, and linearity [6].

The results demonstrate an improvement in the simultaneous detection of these three flavonoids as previous studies described the need for an optimization process or large variations in chromatographic conditions for their convenient separation [41].

#### 3.2.6. Robustness

According to the ICH, robustness should be evaluated as part of the development process before execution of the analytical procedure validation study, and it fundamentally represents the reliability of an analytical procedure after intentional variations in parameters. Regarding HPLC analysis, modifications in the mobile phase are considered adequate to evaluate the method’s capacity to meet the expected performance requirements during normal use [6]. In this study, a slight modification in pH (from 3.32 to 3.43) resulted in a 7.71% decrease in the peak area, although not contributing to a modification in retention time. On the other hand, a slight increase in flow rate (from 1.3 to 1.5 mL/min) resulted in variations in both the peak area (−29.65%) and retention time (−1.3 min). Despite its fast elution of quercetin, this method results in a tailing increase, further compromising peak integration.

#### 3.2.7. Stability

Solution stability is crucial to obtain reliable and reproducible results because a long-run analysis may occur or a repetition of sample analysis may be needed, thus ensuring its integrity throughout time if stored correctly [40].

In this study, three distinct concentration levels were evaluated and submitted to different storage conditions, for different time lengths. The obtained values were compared to the tested concentrations used to determine the accuracy [32].

Concentration variations were calculated for the fifth and seventh day of storage in comparison to the solution preparation day, and it was used to establish their stability. These values were between 68.42% and 122.57% (Table 7).

Following storage at −20 °C, stability ranged between 108.14% and 111.22% after 5 days and 99.29% and 122.57% after 7 days. After storage at 4 °C, the variation was between 108.82% and 112.17% after 5 days and between 105.54% and 111.41% after 7 days. At room temperature, stability was in the range of 85.58% to 110.14% and 68.42% to 105.84% after 5 and 7 days, respectively. Therefore, the best storage condition was at 4 °C, with the solutions remaining stable for 7 days when correctly stored as there was less discrepancy in the stability values compared to the original concentration and amongst themselves. It was also observed that there was greater stability at higher concentrations compared to lower concentrations. Stability results above 100% suggest that solvent evaporation may occur with a consequent increase in quercetin concentration, whereas the observed values below 100% may be justified by the degradation of the analyte.

### 3.3. Overall Assessment of the Method Performance and Quercetin Quantification in Nanoparticles

A comparison of the analytical methods to determine the one that is most fit for purpose may be very challenging owing to the different features that need to be considered. Additionally, consistent bias assessments specifically developed for validation of analytical methods are scarce. Nevertheless, a recently published review considering studies published between 2018 and 2022, performed a bias assessment and concluded about the most fit-for-purpose methods, for quercetin determination. The factors that need to be considered to determine the most suitable method were found to be the retention time, the sensitivity of the methods, and the results of a bias assessment [20]. Regarding retention time, in the present study, a 3.6 min retention time was recorded. Only one of the studies included in the review could provide a more efficient analysis for quercetin, presenting a retention time of 2.8 min [44]. In terms of sensitivity, the method described herein may be considered more sensitive than any of the spectrophotometric methods included in the mentioned review, presenting an LOD of 0.046 µg/mL. In the review, the spectrophotometric method of Srivastava et al. [45], displaying an LOD of 0.33 µg/mL, was considered the most sensitive. With regard to the bias assessment, this study fully satisfied six of the eight parameters: (i) the criteria for acceptable performance was established according to the guidelines, (ii) the reference method using reference material was performed, (iii) the x–y plot of the data was presented, (iv) the difference plot was provided, (v) the regression analysis was considered, and (vi) the linearity test was performed and interpreted; only one of the methods included in the review fully satisfied a maximum of six of the parameters. Accordingly, the present spectrophotometric method developed and validated for the determination of quercetin may be considered superior to the most recent spectrophotometric methods.

The fact that the present method enabled the simultaneous determination of rutin and kaempferol alongside quercetin suggests that it may be used, at least for qualitative purposes, for preparations containing a mixture of the three compounds. The fact that quercetin remained satisfactorily stable at 4 °C for at least seven days after sample preparation is also an important finding that may influence, for example, reanalysis procedures.

The method described herein is currently employed in our laboratory for quercetin detection in nanoparticles.

Polycaprolactone nanoparticles have been used for the improvement of quercetin properties. These nanoparticles are important delivery systems for the treatment of several pathologies once they enable the delivery of compounds in target-specific sites or enable their applications. Therefore, quercetin quantification in this matrix has become useful and imperative.

Quercetin can be detected in a supernatant by evaluating the free compound or after extraction from the nanoparticles. When quercetin is free in a supernatant (water), the preparation of samples consists of the dilution of quercetin in methanol and acetonitrile to obtain a solvent ratio of water/acetonitrile/methanol of 45:15:40. For the extraction from nanoparticles, the nanoparticles are first broken down by dissolving them in methanol assisted by ultrasound and then centrifugated to obtain a quercetin solution, which is then diluted in water and acetonitrile.

Thus, the method’s reproducibility is noteworthy as it can detect and quantify quercetin after using different extraction methods depending on the type of analysis (direct or indirect) and the method used to prepare the standards.

Its selectivity is also notable as no interferents were detected during the analysis. Interferents could eventually appear as a result of the nanoparticle coating or products derived from quercetin that might arise during the production process.

### 3.4. Limitations

Despite different attempts to suppress the existence of a tailing phenomenon in the chromatographic profile of quercetin, it could only be diminished and not fully eliminated. Previous studies have described similar chromatograms when determining quercetin by spectrophotometric detectors and after reviewing analytical methods for this purpose. Mansour et al. [19] concluded that tailing is a frequent incident when using reverse-phase chromatography. Tailing was depicted in quercetin chromatograms of methods developed for the analysis of *Bauhinia variegata* Linn flower extract [46], German grape wines [47], *Ginkgo biloba* leaf extract [48], *Costus igneus* leafs [49], *Hibiscus sabdariffa* L. calyces [50], and *Brassica oleracea* L. var. *capitata* plant material [51]. Particularly regarding reverse-phase chromatography methods like the one described herein, tailing is known to be due to “mixed-mode” retention, where the hydrophobic monolayer comprises one type of site and the “active” silanols comprise a second type of site [52]. Although commonly described, it is important to acknowledge that the tailing phenomenon is undesirable, may compromise the quantification accuracy, and constitutes an important limitation to the present study.

Although the presently described HPLC-DAD method has proved to be fit for the analysis of quercetin in pharmaceutical preparations, the adequacy for determination of quercetin in complex matrices—both from biological matrices and from natural sources—is unknown. It is important to acknowledge that the complexity of those matrices may increase noise in the chromatogram and result in coeluting peaks. For more complex matrices and especially if low analyte levels are to be expected, mass spectrometric methods may be more appropriate given their increased sensitivity. Recently, mass spectrometric methods for the detection of quercetin in complex matrices have been used for the analysis of samples of *Ziziphus jujuba* and *Ziziphus nummularia* plants [53], berries [54], and *Polygonatum verticillatum* rhizomes [55].

## 4. Materials and Methods

### 4.1. Reagents

Methanol (≥99.8%; 32.04 g/mol) and acetonitrile (≥99.9%; 41.05 g/mol) were purchased from Fisher Scientific (Loughborough, UK). Acetic acid (60.05 g/mol) was purchased from VWR^®^ (Fontenay-sous-Bois, France). Quercetin (≥95.0%; 302.24 g/mol) and rutin (≥94.0%; 610.52 g/mol) were purchased from Sigma-Aldrich^®^ Co. (St. Louis, MO, USA). Kaempferol (≥98.0%; 286.24 g/mol) was purchased from Santa Cruz Biotechnology, Inc. (Dallas, TA, USA).

### 4.2. Chromatographic Conditions

HPLC analysis was conducted on a JASCO^®^ equipment with a reverse-phase C-18 column (LiChroCART^®^ 250-4, LiChrosorb^®^, RP-18, 250 mm × 4 mm, 5 µm), a DAD (MD-4010, Jasco^®^, Oklahoma City, OK, USA), an autosampler (AS-4050, Jasco^®^, Oklahoma City, OK, USA), and a pump (PU-4180, Jasco^®^, Oklahoma City, OK, USA). ChromNav^®^ 2.0 was the software employed for data acquisition and analysis, which was set at room temperature with an injection volume of 10 µL. Detection was performed at 254 and 368 nm. Different mobile phase mixtures were evaluated in an isocratic elution. Previously purified water was filtered with a 0.22 µm nylon filter, and all solvents were degassed by ultrasound. A ratio of 55:40:5 of water/acetonitrile/methanol, acidified with 1.5% acetic acid, was used as the mobile phase. The flow rate was as follows: 1.000 (0–3.5 min), 1.300 (3.5–8 min), and 1.000 (8 min until analysis finale) mL/min. The chromatographic run was set for 10 min. 

### 4.3. Standard Solution Preparation

Quercetin stock solution (0.49 mg/mL) was prepared in a water/acetonitrile/methanol ratio of 45:15:40. Then, successive dilutions were made to obtain the standard solutions. Rutin and kaempferol solutions were obtained following the same procedure. 

### 4.4. Method Validation

#### 4.4.1. Linearity and Range

The linearity of the analytical method optimized for quercetin quantification was determined using nine quercetin standard solutions (0.14, 0.35, 0.57, 2.8, 5, 65, 125, 185, and 245 µg/mL) [6,32]. Analysis was performed over different days in triplicate, and a calibration line comprising all analyses was constructed.

One of the strategies to evaluate linearity is the RSD of the slope [6,38]. Accordingly, RSD was calculated using Equation (1):

RSD (%) = s_m_/m × 100 (1)

where “s_m_” represents the slope standard deviation and “m” represents the slope.

#### 4.4.2. Limit of Detection and Limit of Quantification

As recommended, LOD and LOQ were determined based on the calibration curve (specifically, using the calibration curve slope) and standard deviation of the response obtained through the analysis of 10 blank samples analyzed in triplicate [6,56]. These parameters were determined employing Equations (2) and (3), respectively [6]:

LOD = 3.3 × δ/s(2)

LOQ = 10 × δ/s(3)

where “δ” represents the standard deviation of the response, and “s” represents the calibration curve slope.

#### 4.4.3. Precision

Intra- and interday precisions were calculated by analyzing quercetin standard solutions in five distinct concentrations (0.35, 0.57, 5, 125, and 185 µg/mL) in triplicate and subsequently expressed under RSD [6] (Equation (4)):

RSD (%) = s/X × 100(4)

where s represents the standard deviation of a series of measurements, and X represents the mean value from the independent variable.

#### 4.4.4. Accuracy

Regarding the method’s accuracy determination, six quercetin standard solutions with known concentrations (0.35, 0.49, 0.57, 49, 125, and 196 µg/mL) were injected in triplicate for subsequent backcalculation of the area values obtained in the calibration equation, and further comparison of the concentration was carried out with the values of the actual concentrations [6] through Equation (5) (n = 5):

Accuracy = Experimental value/Real value × 100(5)

#### 4.4.5. Specificity/Selectivity

Different solutions were prepared with two other compounds whose chemical structure was similar to quercetin and which can be found in several matrices, together with quercetin, such as medicinal plants: rutin and kaempferol [57]. Solutions containing 0.245 µg/mL of each of the three compounds were prepared and analyzed in triplicate to verify the ability to separate the peaks of the different compounds under analysis [6].

#### 4.4.6. Robustness

Robustness was assessed in relation to slight variations in two parameters of the mobile phase, as suggested by the ICH guidelines: pH and flow rate [6]. Thus, a 1.1 increase in pH and an increase of 0.2 mL/min in flow rate were both individually studied and compared to the optimized method. The differences regarding peak area and retention time were determined.

#### 4.4.7. Stability

Three concentrations of standard solutions (0.57, 5, and 125 µg/mL) were analyzed in three different storage conditions (−20 °C, 4 °C, and room temperature) at different times (5 and 7 days postpreparation) to determine the stability of the solutions prepared for analysis [5]. Stability was calculated by comparison with the day of its preparation, and variations in the results were determined using Equation (6):

Stability (%) = Concentration (day x)/Concentration (day 0) × 100(6)

## 5. Conclusions

A simple, rapid, accurate, and precise method was successfully optimized and validated following existing guidelines for quercetin detection and quantification. The method comprised a wide concentration range with the application of adjusted equations for quantification depending on the concentration of quercetin after first analyzing using a nonadjusted equation. Quercetin was rapidly eluted, enabling a short analysis time and, consequently, a more ecologic and economic approach than most previously reported methods. This may be a promising method for analysis of quercetin contained in nanoparticles for new, effective, and safe biological and therapeutic approaches. Given the sensitivity of the method, its efficiency, and the fulfilment of most bias assessment parameters, it stands as a particularly convenient method compared to most recently published studies using the HPLC-DAD technology, which is available widespread, for quercetin determination. This method will be evaluated in the future for its suitability for the determination of quercetin in more complex matrices and the possible inclusion of additional analytes.

## Figures and Tables

**Table 1:** Linearity range, regression equation, determination coefficient, and correlation coefficient of quercetin.

Compound—Calibration Curve	Linearity Range (µg/mL)	Calibration Equation	r^2^	r
QUE—nonadjusted	0.14–245	y = 15,485x + 11,659	0.9976	0.9988
QUE—adjusted [1]	0.14–5	y = 15,262x − 271.48	0.9994	0.9997
QUE—adjusted [2]	5–245	y = 15,317x + 42,292	0.9954	0.9977

QUE: quercetin; r: correlation coefficient; r^2^: determination coefficient.

**Table 2:** Backcalculation of quercetin standards with deviations.

QUE Concentration (µg/mL)	QUE—Nonadjusted		QUE—Adjusted [1]		QUE—Adjusted [2]	
	[Obtained]	Accordance (%)	[Obtained]	Accordance (%)	[Obtained]	Accordance (%)
0.14	−0.62	−442	0.15	107	-	-
0.35	−0.37	−106	0.41	117	-	-
0.57	−0.29	−51	0.49	86	-	-
2.8	1.98	71	2.8	100	-	-
5	4.16	83	5	100	2.2	44
65	64.25	100	-	-	63	97
125	130.06	104	-	-	129	103
185	193.26	104	-	-	193	104
245	236.40	96	-	-	234	96

[Obtained]: obtained concentration; QUE: quercetin.

**Table 3:** Data from the analysis of intercept of the ordinate at origin and relative standard deviation of the slope criteria for linearity evaluation.

Compound—Calibration Curve	m	s_m_	Slope RSD (%)	95% Confidence Interval	
				Minimum	Maximum
QUE—nonadjusted	15,485	289.47	1.87	−65,423	88,741
QUE—adjusted [1]	15,262	211.13	1.38	−2006	1463
QUE—adjusted [2]	15,317	601.69	3.93	−247,002	331,585

QUE: quercetin; m: slope; s_m_: slope standard deviation; RSD: relative standard deviation.

**Table 4:** Analytical data for precision (intra- and interday) of quercetin.

QUE Concentration (µg/mL)	Intraday Precision—RSD (%)	Interday Precision—RSD (%)
0.35	5.66	9.42
0.57	5.47	8.19
5	6.74	6.87
125	2.41	7.38
185	2.64	7.18

Values were obtained from five independent samples (n = 5) in triplicate; QUE: quercetin; RSD: relative standard deviation.

**Table 5:** Analytical data for accuracy of quercetin.

QUE Concentration (µg/mL)	Obtained Concentration	Accuracy (%)
0.35	0.31	88.6
0.49	0.51	104.1
0.57	0.52	91.2
49	52	106.1
125	121	96.8
196	217	110.7

QUE: quercetin.

**Table 6:** Analytical data for robustness evaluation regarding pH and flow rate parameters.

Method	Parameter	Parameter Value	Peak Area	% of Peak Area in Relation to Optimized Method	Retention Time (min)	Difference of Retention Time in Relation to Optimized Method (min)
Optimized	pH	3.32	7,304,837	N/A	3.8	N/A
variation for robustness evaluation		3.43	6,741,931	92.29	3.8	0.0 min
Optimized	Flow rate	1.3 mL/min	6,111,868	N/A	3.7	N/A
variation for robustness evaluation		1.5 mL/min	4,299,546	70.35	2.4	−1.3 min

N/A: Not applicable.

**Table 7:** Analytical data for stability of quercetin after 5 and 7 days in three storage conditions (−20 °C, 4 °C, and room temperature) for three levels of concentration.

Condition	Concentration (µg/mL)	Day 0	Day 5		Day 7	
		Mean (n = 3)	Mean (n = 3)	Stability (%)	Mean (n = 3)	Stability (%)
−20 °C	0.57	7129	7929	111.22	8737	122.57
	5	71,284	78,463	110.07	77,477	108.69
	125	2,170,741	2,347,331	108.14	2,155,391	99.29
4 °C	0.57	8048	8758	108.82	8697	111.41
	5	74,799	83,905	112.17	78,945	105.54
	125	2,123,647	2,318,601	109.18	2,255,046	106.19
Room temperature	0.57	7799	6674	85.58	5336	68.42
	5	73,338	75,772	103.32	67,525	92.07
	125	2,246,811	2,474,717	110.14	2,378,043	105.84

## Data Availability

Data is contained within the article and Supplementary Material.

## Supplementary Materials

The following supporting information can be downloaded at: https://www.mdpi.com/article/10.3390/ph16121736/s1, Figure S1: Calibration curve of quercetin—nonadjusted for a linear range of 0.14–245 µg/mL; Figure S2: Calibration curve of quercetin. a: Quercetin—adjusted [1] for a linear range of 0.14–5 µg/mL. b: Quercetin—adjusted [2] for a linear range of 5–245 µg/mL; Figure S3: Difference plot of concentrations between 0.14 and 5 µg/mL, exhibiting the variance between the linear theoretical range (discontinuous line), backcalculation of the concentration given by the nonadjusted calibration curve (orange dots), and backcalculation of the concentration given by the adjusted calibration curve (gray dots); Figure S4: Difference plot of concentrations between 5 and 245 µg/mL, exhibiting the variance between the linear theoretical range (discontinuous line), backcalculation of the concentration given by the nonadjusted calibration curve (orange dots), and backcalculation of the concentration given by the adjusted calibration curve (gray dots).
