# Supplementary figures and images for: Validation of an HPLC-DAD Method for Quercetin Quantification in Nanoparticles
Source: Pharmaceuticals (Basel). 2023 Dec 17;16(12):1736. doi: 10.3390/ph16121736 (PMC10748265; doi:10.3390/ph16121736)

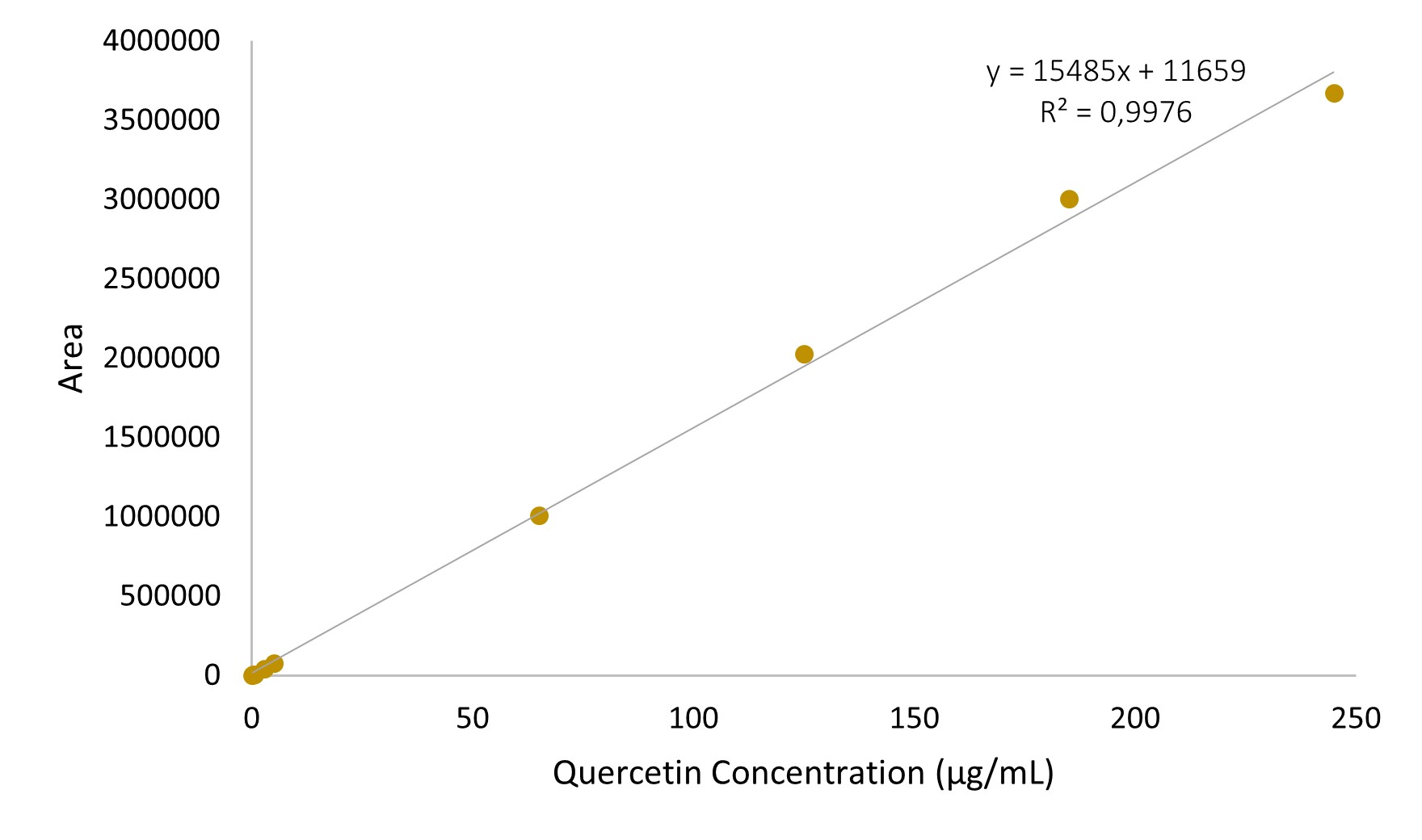

Supplement: Supplementary file 1 [file pharmaceuticals-16-01736-s001.zip › Figure S1.jpg]

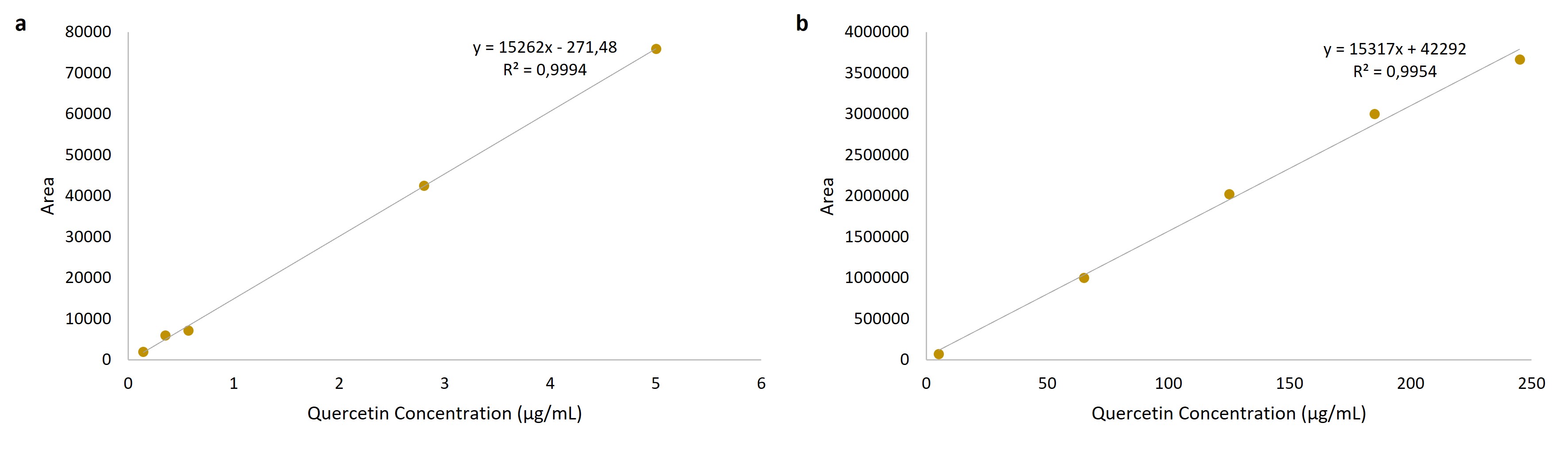

Supplement: Supplementary file 1 [file pharmaceuticals-16-01736-s001.zip › Figure S2.jpg]

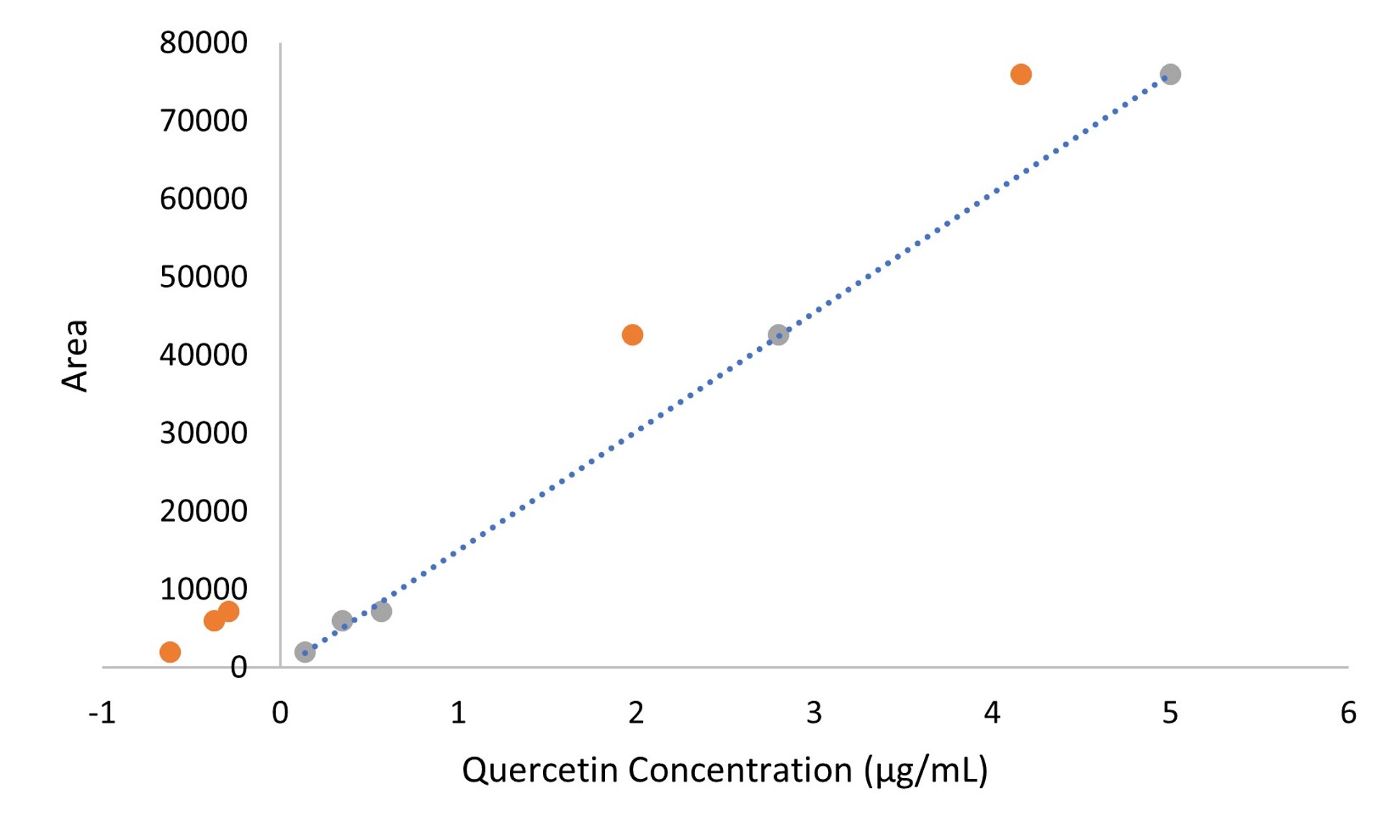

Supplement: Supplementary file 1 [file pharmaceuticals-16-01736-s001.zip › Figure S3.jpg]

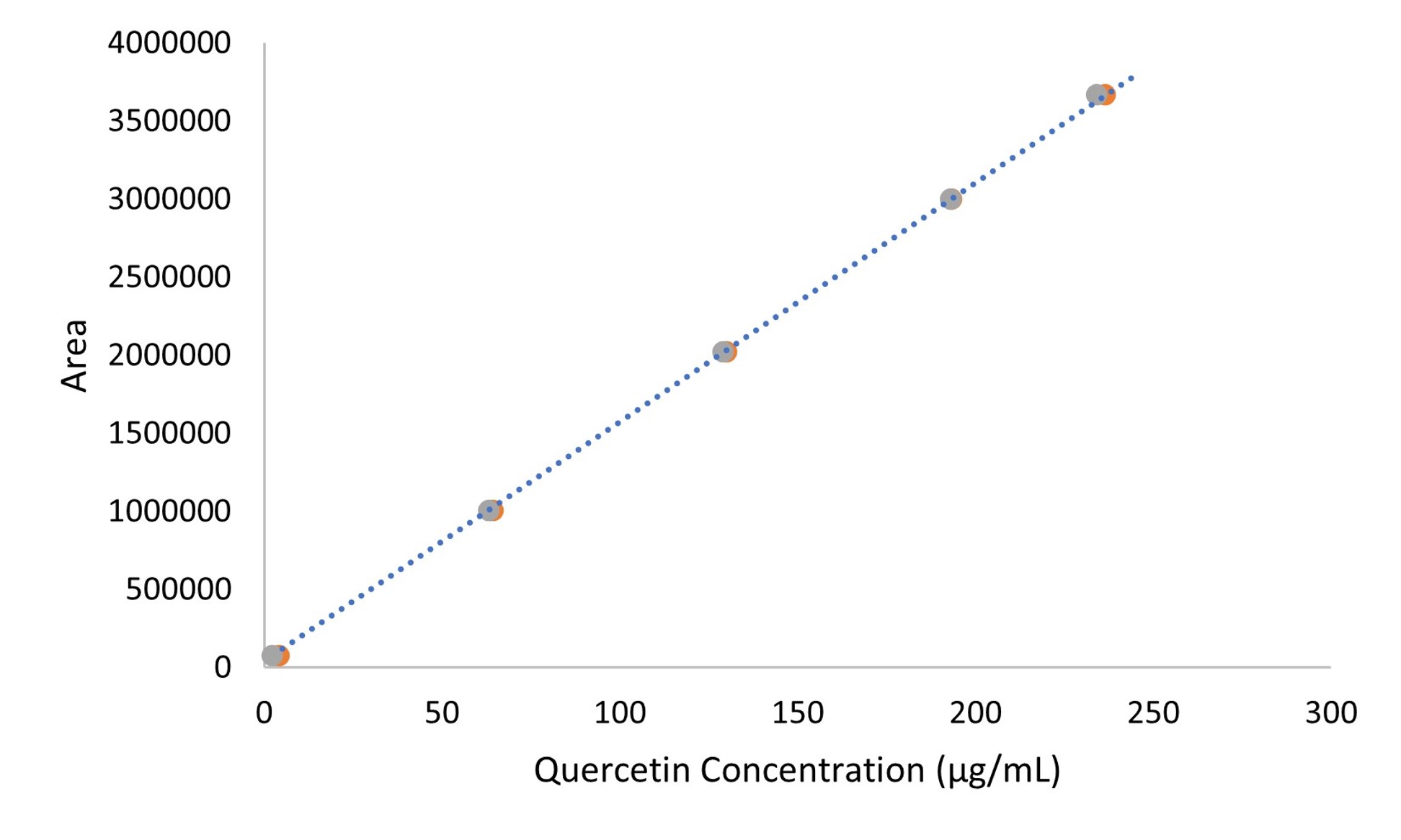

Supplement: Supplementary file 1 [file pharmaceuticals-16-01736-s001.zip › Figure S4.jpg]
